# 

**Published:** 1893-09

**Authors:** 


### CONTENTS.

ORIGINAL COMMUNICATIONS—
The Practice of Medicine in Georgia. By Arthur C. Blain, B. C. S., M. D., Macon, Ga. .... 385
Ante and Retro-Positions of the Uterus—Their Pathology, Symptomatology and Treatment. By W. W. Stewart, M. D., Columbus, Ga. .... 390
Hemorrhagic Fever. By O. B. Bush, M. D., Arlington, Ga. .... 396
The Local Treatment of Psoriasis. By R. C. Longfellow, M. D., Cincinnati. .... 398
SOCIETY REPORTS—
Philadelphia Academy of Surgery. .... 402
Transactions of the Atlanta Obstetrical Society. .... 409
CORRESPONDENCE—
A Monument to the Memory of the Late Dr. Willis F. Westmoreland, Sr. .... 416
EDITORIAL—
Yellow Fever. .... 417
The Examining Board and the Public Press. .... 420
SELECTIONS AND ABSTRACTS—
Surgery. .... 422
Obstetrics and Gynecology. .... 429
Skin Diseases and Syphilis. .... 434
General Medicine and Therapeutics. .... 438
Eye, Ear, Nose and Throat. .... 441
MEDICAL ITEMS. .... 444
BOOK REVIEWS. .... 446
ADVERTISERS' INDEX TO ADVERTISEMENTS. .... xii
CLASSIFIED INDEX TO ADVERTISEMENTS. .... xlv
ITEMS OF INTEREST. .... xi
PREMIUM OFFERS. .... xv

